# A pathogenic in-frame deletion-insertion variant in *BEST1* phenocopies Stargardt disease

**DOI:** 10.1172/jci.insight.162687

**Published:** 2022-12-08

**Authors:** Masha Kolesnikova, Jin Kyun Oh, Jiali Wang, Winston Lee, Jana Zernant, Pei-Yin Su, Angela H. Kim, Laura A. Jenny, Tingting Yang, Rando Allikmets, Stephen H. Tsang

**Affiliations:** 1 Jonas Children’s Vision Care and Bernard and Shirlee Brown Glaucoma Laboratory, Columbia University, New York, New York, USA.; 2 SUNY Downstate Health Sciences University, New York, New York, USA.; 3 Department of Ophthalmology,; 4 Department of Genetics and Development, and; 5 Department of Pathology and Cell Biology, Columbia University, New York, New York, USA.; 6 Institute of Human Nutrition, Columbia Stem Cell Initiative, New York, New York, USA.

**Keywords:** Genetics, Ophthalmology, Genetic diseases, Retinopathy

## Abstract

Here, we describe affected members of a 2-generation family with a Stargardt disease–like phenotype caused by a 2–base pair deletion insertion, c.1014_1015delGAinsCT;p.(Trp338_Asn339delinsCysTyr), in *BEST1*. The variant was identified by whole-exome sequencing, and its pathogenicity was verified through chloride channel recording using WT and transfected mutant HEK293 cells. Clinical examination of both patients revealed similar phenotypes at 2 different disease stages that were attributable to differences in their age at presentation. Hyperautofluorescent flecks along the arcades were observed in the proband, while the affected mother exhibited more advanced retinal pigment epithelium (RPE) loss in the central macula. Full-field electroretinogram testing was unremarkable in the daughter; however, moderate attenuation of generalized cone function was detected in the mother. Results from electrooculogram testing in the daughter were consistent with widespread dysfunction of the RPE characteristic of Best disease. Whole-cell patch-clamp recordings revealed a statistically significant decrease in chloride conductance of the mutant compared with WT cells. This report on a mother and daughter with a *BEST1* genotype that phenocopies Stargardt disease broadens the clinical spectrum of *BEST1*-associated retinopathy.

## Introduction

*BEST1*, located on chromosome 11q13, encodes the bestrophin-1 protein, a transmembrane calcium-sensitive chloride channel located in the retinal pigment epithelium (RPE) (1). The alteration of bestrophin results in fluid and lipofuscin accumulation beneath the retina, leading to serous neurosensory detachment and secondary degeneration of photoreceptors (2). Nearly 500 variants have been identified in the *BEST1* gene; these variants lead to a wide variety of phenotypic associations collectively known as the bestrophinopathies, which include adult-onset vitelliform macular dystrophy, autosomal dominant vitreoretinochoroidopathy, both autosomal recessive and autosomal dominant bestrophinopathy, bull’s eye maculopathy, retinitis pigmentosa, and microcornea, rod-cone dystrophy, cataract, posterior staphyloma syndrome (1, 3).

Best vitelliform macular dystrophy (BVMD), also known as Best disease, is an autosomal dominant inherited retinal dystrophy that occurs in roughly 1 in 15,000–20,000 individuals (4). There are multiple reports of incomplete penetrance in BVMD (5–7). Typically, BVMD presents early in life with fundus findings of vitelliform, or egg yolk-like lesions, that later progress to RPE atrophy; however, the age of onset can be variable, with cases of disease onset as late as 75 years old (8). These patients will often have unperturbed visual acuity early on (2), and up to 5% of patients with a genetic diagnosis of BVMD never develop symptoms or typical fundus findings (8). At more advanced stages, visual deterioration occurs along with the onset of metamorphopsia (2). Nonetheless, visual acuity is often preserved in at least one eye throughout the natural history of disease progression (8). The most sensitive diagnostic test for BVMD is the electrooculogram (EOG). It will show diminished light rise before the onset of ocular symptoms, resulting in a decreased Arden ratio, which will even be abnormal in patients without evident fundus findings (2, 3, 9). A full-field electroretinogram (ffERG), which is frequently used to diagnose other retinal dystrophies, is normal in patients with BVMD.

Given the significant genetic variability responsible for the bestrophinopathies, it has been previously suggested that it is difficult to assess genotype-phenotype correlations; however, others have remained more optimistic (2). To date, a wide variety of clinical presentations have been associated with pathogenic variation in *BEST1*, and these continue to expand. This report describes what we believe to be a novel phenotype of the *BEST1* variant, c.1014_1015delGAinsCT;p.(Trp338_Asn339delinsCysTyr), as seen in a mother and daughter with BVMD phenocopying Stargardt disease.

## Results

### Case description.

**Figure 1:**
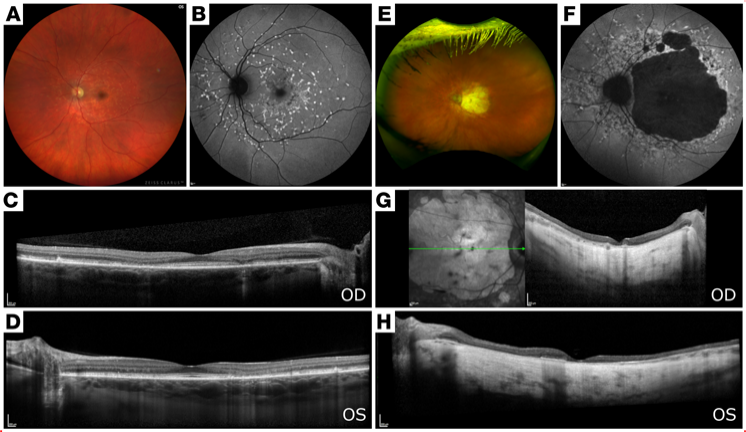
Yellow pisciform flecks in a mother and daughter with an in-frame deletion-insertion variant in *BEST1*, as seen using fundus photography and spectral-domain optical coherence imaging. (**A**) Color fundus image of the left eye of a 37-year-old woman, showing central hyperpigmentation in the fovea and yellow pisciform flecks along the arcades with sparing of the central fovea. (**B**) Short-wave fundus autofluorescence (SW-FAF) imaging of the left eye, demonstrating a similar pattern of hyperautofluorescent flecks along the arcades, extending centrally toward the macula as well as several hypoautofluorescent lesions surrounding the fovea bilaterally. (**C** and **D**) Spectral domain optical coherence tomography (SD-OCT) imaging, showing parafoveal retinal thinning of the outer nuclear layers and attenuation of the ellipsoid zone. (**E**) Color fundus image of the left eye of the 69-year-old mother of the person in **A**, showing substantial bilateral retinal pigment epithelium loss in the central macula up to the arcades and relative peripapillary sparing. (**F**) SW-FAF imaging of the left eye, revealing extensive macular atrophy with a surrounding pattern of fleck extending into the periphery. (**G** and **H**) SD-OCT imaging, showing extensive loss of retinal architecture and extensive retinal atrophy with hypertransmission into the choroid.

**Figure 2:**
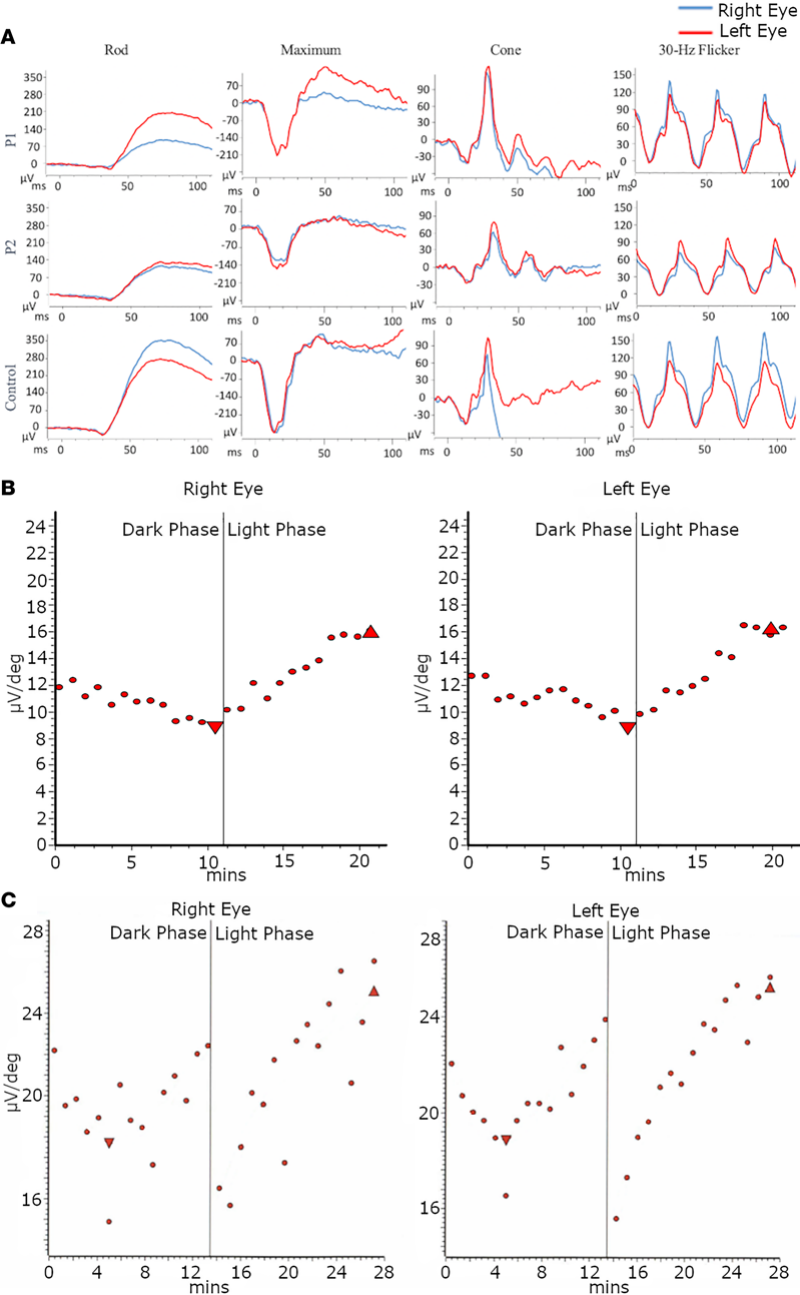
Electrooculogram and full-field electroretinogram testing results from a mother and daughter with an in-frame deletion-insertion variant in *BEST1*. (**A**) Full-field electroretinogram (ffERG) of the daughter (P1) revealed fully preserved scotopic rod-specific, maximum, and photopic cone single flash and 30 Hz flicker responses. ffERG of the mother (P2) revealed that scotopic rod-specific response ERG b-wave amplitudes were 133 microvolts in the right eye and 155 microvolts in the left eye. Maximal ERG a-wave and b-wave amplitudes were 94 and 84 microvolts in the right eye and 120 and 117 microvolts in the left eye. Transient photopic ERG b-wave amplitudes and implicit times were 87 microvolts and 32 milliseconds in the right eye, 106 microvolts and 33 milliseconds in the left eye. Photopic 30 Hz flicker ERG had implicit times and amplitudes of 77 microvolts and 30 milliseconds in the right eye, 99 microvolts and 31 milliseconds in left eye. A normal 43-year-old control is shown for comparison. (**B**) Electrooculogram (EOG) of the daughter, showing diminished light rise bilaterally with Arden ratios of 1.51 and 1.47 in the right and left eyes, respectively. (**C**) EOG of the mother was affected by poor fixation but showed diminished light rises, with Arden ratios of 1.28 in the right eye and 1.34 in the left eye.

A 37-year-old woman (P1) presented to the medical retina clinic at the Columbia University Irving Medical Center for evaluation, with a referring diagnosis of Stargardt disease. The patient was asymptomatic, stating that she was referred based on incidental findings seen during a routine dilated fundus examination. The patient reported a family history of age-related macular degeneration (AMD) in her mother, maternal uncle, and maternal grandfather. At initial presentation, her visual acuity was best corrected to Snellen 20/20 in both eyes. Anterior segment examination was unremarkable. Dilated fundus examination revealed a pattern of yellow pisciform flecks across the macula, sparing the juxta-papillary region in both eyes. The foveal region exhibited a hyperpigmented appearance but was otherwise healthy (Figure 1A). Short-wavelength autofluorescence (SW-AF) revealed hyperautofluorescent flecks along the arcades, extending centrally toward the macula with peripapillary sparing (Figure 1B). Rare central hypoautofluorescent lesions were seen surrounding the fovea bilaterally. Spectral domain–optical coherence tomography (SD-OCT) revealed parafoveal outer retinal atrophy and abrupt disruption of the photoreceptor-attributable ellipsoid zone band in the right eye and retinal thinning of the outer nuclear layers temporal to the fovea, with attenuation of the ellipsoid zone in the left eye (Figure 1, C and D). Axial lengths were measured to be 24.09 mm in the right eye and 24.22 mm in the left eye. ffERG testing showed no generalized rod and cone dysfunction in both eyes (Figure 2A). EOG showed diminished light rise bilaterally, with an Arden ratio of 1.51 in the right eye and 1.47 in the left (Figure 2B).

The affected mother (P2) was a 69-year-old woman previously diagnosed with age-related AMD at 45 years of age. Her visual acuity was best corrected to count fingers at 3 feet in the right eye and 20/250 in the left eye. She reported a family history of AMD in her father and brother who experienced an onset of visual symptoms at ages 60 and 45 years, respectively. Anterior segment examination was remarkable for trace nuclear sclerosis (NS +1) in both eyes. Dilated fundus examination revealed a substantial area of bilateral RPE loss in the central macula extending to the arcades (Figure 1E). SW-AF revealed extensive macular atrophy, with a surrounding pattern of flecks extending in the periphery and relative peripapillary sparing (Figure 1F). SD-OCT showed profound thinning, extensive loss of retinal architecture, and complete outer retinal atrophy with hypertransmission in the choroid (Figure 1, G and H). ffERG revealed relatively unaffected dark-adapted rod-specific responses, with a low B-to-A ratio on maximum responses bilaterally (Figure 2A). Single flash cone and 30 Hz flicker responses showed preserved amplitudes without implicit time delay in either eye. EOG, although affected by poor fixation, revealed diminished light rise bilaterally, with reduced Arden ratios of 1.28 and 1.34 in the right and left eyes, respectively (Figure 2C).

### Exome sequencing analysis.

Whole-exome sequencing was performed in the proband, and variant filtering was restricted to nonsynonymous exonic and canonical splice site variants in genes previously associated with retinal disease (RetNet; https://sph.uth.edu/retnet/, accessed May 2022). Fifteen heterozygous variants were identified with a minor allele frequency (MAF) equal to or less than 0.005 (Table 1). Rare and predicted pathogenic variants were identified in several genes, including a canonical splice site variant, c.97-2A > G (SpliceAI Δscore =1.00), in *NEK2*, missense variants in *PLK4*, *EYS*, *RPGRIP1L*, *DMD*, and *BEST1*; however, all except the latter, are associated with autosomal or X-linked recessive retinal diseases. Interestingly, no pathogenic variant was identified in the *ABCA4* gene, the causal gene for Stargardt disease (Supplemental Table 1; supplemental material available online with this article; https://doi.org/10.1172/jci.insight.162687DS1).

The *BEST1* (NM_004183.4) variant is a 2 bp in-frame deletion-insertion (c.1014_1015delGAinsCT;p.(Trp338_Asn339delinsCysTyr)) of 2 highly conserved nucleotides (ref. 10; phyloP100way > 9) that code for 2 residues in the intracellular domain of the protein. The variant is absent from the general population (i.e., ultrarare) according to the gnomAD database and is predicted deleterious by deletion-insertion–specific pathogenicity algorithms: MutPred-InDel (ref. 11; g = 0.63) and PROVEAN (ref. 12; score = –18.81). When analyzed as individual variants, the p.(Trp338Cys) and p.(Asn339Tyr) substitutions are also universally predicted to be highly pathogenic across SNV-specific algorithms. Direct sequencing of exon 9 of *BEST1* in the affected mother confirmed maternal segregation of this variant. In the proband, no putatively pathogenic *ABCA4* variants were identified at a MAF filter of less than or equal to 0.005. Several intronic, synonymous, and missense *ABCA4* variants present at an MAF threshold of less than or equal to 0.1 (Supplemental Table 1); although, as expected, none are predicted to have any contributing effect in this case.

### Functional testing of BEST^Trp338_Asn339delinsCysTyr^.

**Figure 3:**
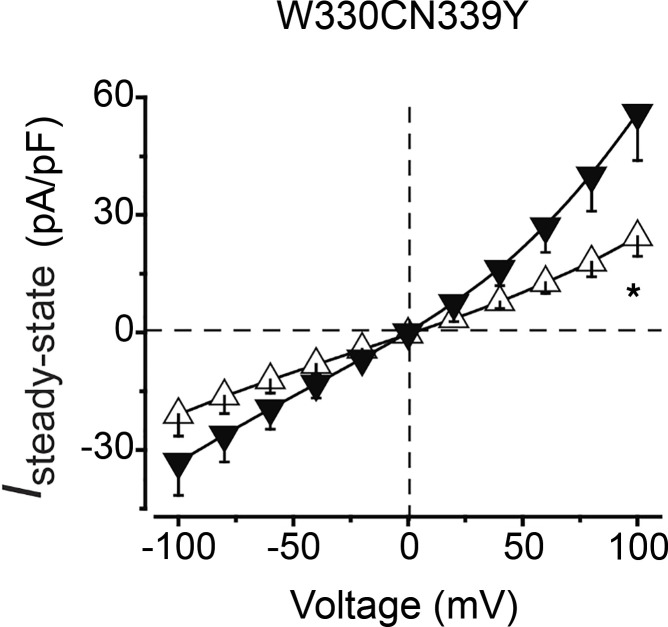
Diminished chloride conductance in cells expressing Trp338_Asn339delinsCysTyr. Population steady-state current density-voltage relationships at 1 μM Ca^2+^ in HEK293 cells expressing WT (black) and mutant (white) human *BEST1*. *n* = 5–6 for each point. *P < 0.05 compared with currents from WT, using 2-tailed unpaired Student’s *t* test.

Electrophysiological analyses of whole-cell patch-clamp recordings revealed decreased density-voltage relationships between HEK293 cells expressing WT and mutant human *BEST1* (Figure 3). Two-tailed unpaired Student’s *t* tests between WT and mutant HEK293 cells suggested significant differences in chloride conduction (*P* < 0.05).

## Discussion

The variability in phenotype has been a topic of interest, with many reports working to assess a genotype-phenotype correlation and documenting unusual presentations, such as those with widespread flecks in the midperiphery (8) and bone spiculation in the far periphery reminiscent of retinitis pigmentosa (13). This case report further expands the known phenotypes of *BEST1* by describing a mother and daughter who were found to have genetic diagnosis of *BEST1*, c.1014_1015delGAinsCT;p.(Trp338_Asn339delinsCysTyr) and presented with fundus findings of extensive pisciform fleck and central macular atrophy phenocopying Stargardt disease. This variant causes the substitution of 2 highly conserved cytoplasmic bestrophin residues (14) and was shown to be pathogenic in our functional modeling. *BEST1* functions selectively as a calcium-gated chloride channel within the RPE, and targeted testing of chloride conductance revealed decreased conductance in transfected HEK293 cells in comparison with WT cells. This was more suggestive of a loss-of-function mutation as opposed to gain of an alternative function.

This rare variant has been described only once previously in a compound heterozygous patient with a diagnosis of autosomal recessive bestrophinopathy (ARB) (15). A number of BEST1 variants, such as the c.884_886delTCA;p.(Ile295del) (15) and c.422G > A;p.(Arg141His) (16, 17) variants, have been associated with both BVMD and ARB. We suggest that the c.1014_1015delGAinsCT;p.(Trp338_Asn339delinsCysTyr) variant may be included in the list of variants associated with both BVMD and ARB.

Numerous inherited retinal dystrophies that phenotypically mimic Stargardt disease have been previously reported, including those associated with variants in *ELOVL4*, *PROM1*, *RDS/PRPH2*, and *CLN3* (18). Variants in *BEST1* have been frequently suggested to also be responsible for a Stargardt disease–like degeneration, owing to a shared pathophysiology (19–21). However, there have been only two prior reports of *BEST1* presenting with fleck-like lesions: a 54-year-old man with fleck nasal to the disc and along the temporal arcades (9) and a 74-year-old-man presenting with midperipheral fleck late in life (8).

**Figure 4:**
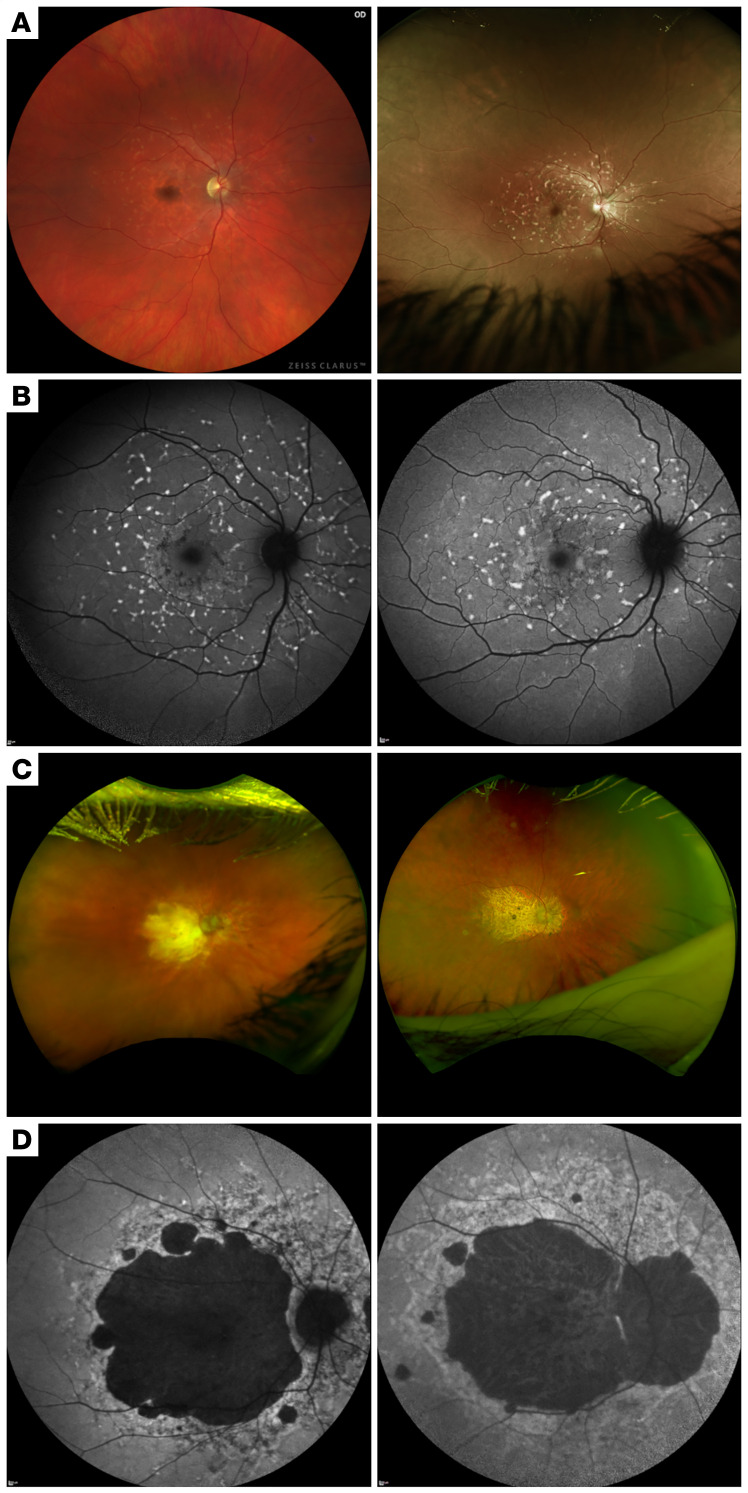
*BEST1*^Trp338_Asn339delinsCysTyr^ phenocopies Stargardt disease on fundus images and fundus autofluorescence imaging. (**A**) A color fundus image of a 37-year-old woman with BVMD (left) and a color fundus image of a 45-year-old woman with a confirmed diagnosis of Stargardt disease (right), revealing similar patterns of yellow pisciform flecks, extending anterior to the arcades and nasal to the optic disc. (**B**) Short-wavelength imaging similarly shows similar patterns of hyperautofluorescent flecks along the arcades, extending centrally toward the macula between a 69-year-old woman with BVMD (left) and a patient with Stargardt disease (right). (**C**) Color fundus image and (**D**) short-wavelength autofluorescence imaging of a 69-year-old woman with BVMD (left) and images of an 80-year-old man with severe Stargardt disease (right), demonstrating severe macular atrophy approaching the arcades.

P1 presented with fundus findings of yellow pisciform flecks extending anterior to the arcades and nasal to the optic disc, reminiscent of a Stargardt disease phenotype (Figure 4A) (18). The SW-AF images of P1 showed a similar pattern to the SW-AF pattern seen in Fishman group II Stargardt disease, demonstrating hyperautofluorescent flecks along the arcades, extending centrally toward the macula (Figure 4B) (22). Although the clinical presentation was consistent with Stargardt disease, both CLIA and extensive research genetic testing revealed no variants in *ABCA4* or other known phenocopies, and EOG results revealed an abnormal light rise consistent with BVMD. ffERG revealed normal scotopic rod-specific and photopic cone-specific responses, which would be expected in both Stargardt disease and BVMD (23). In contrast, P2 presented with a fundus phenocopy of a more advanced Stargardt disease phenotype, likely attributable to the difference in age between the patients. The fundus findings revealed severe macular atrophy, extending to the arcades, and SW-AF demonstrated similar patterns of macular atrophy invading the arcades, comparable to Fishman group III, between P2 and a patient with a confirmed diagnosis of Stargardt disease (Figure 4, C and D) (22). This patient was also found to have the same heterozygous variant in *BEST1*. EOG, although affected by the patient’s poor fixation, showed diminished light rise bilaterally, and ffERG revealed preserved scotopic rod-specific responses with a low B-to-A ratio, suggesting inner retinal dysfunction consistent with that in published literature (24–26) and diminished photopic-cone single flash and 30 Hz flicker responses. While ffERG findings are often normal in early BVMD and Stargardt disease, diminished scotopic and photopic responses can be expected in later-stage Stargardt disease (23). Only patients diagnosed with multifocal BVMD, the autosomal recessive form of the disease (27), will show abnormal ffERG results (28, 29).

A possible explanation for the phenotypic similarity between the BVMD diagnosed in these patients and Stargardt disease lies in the histopathologic findings. Petrukhin et al. (21) have shown that both BVMD and Stargardt disease are associated with lipofuscin accumulation, and others have similarly suggested that BVMD may share a pathophysiology with Stargardt disease (30–32). Lipofuscin is a complex aggregate of cellular components that is found in various cells throughout the body; in the RPE specifically, lipofuscin accumulation results from phagocytosis of the photoreceptor outer segments (33). Extensive study of the lipofuscin accumulation in BVMD has demonstrated increased levels of A2E, the same component of lipofuscin that has been found in high levels in Stargardt disease (30, 31). Although the exact mechanism by which A2E accumulation occurs in BVMD is unknown, it has been suggested that impairment of the bestrophin calcium-sensitive chloride channels leads to loss of both phagolysosome acidification and regulation of vesicle fusion, either of which may cause the subsequent accumulation of lipofuscin (30).

Variants in *BEST1* are a well-described cause of inherited retinal dystrophy, with a continually expanding variety of phenotypes. In this report, we described the case of a mother and daughter presenting with BVMD phenocopying Stargardt disease caused by a heterozygous pathogenic variant in *BEST1*. Given the possible novelty of the variant and the phenotype, future studies of the functional impact of this variant as well as additional genotype-phenotype correlation in larger cohorts may help clarify the similarity in disease pathophysiology between BVMD and Stargardt disease.

## Methods

### Clinical evaluation.

Two patients were evaluated at the Edward S. Harkness Eye Institute at the Columbia University Medical Center. Patients underwent complete ophthalmic examination, beginning with dilation using topical tropicamide (1%) and phenylephrine hydrochloride (2.5%). Imaging studies were conducted using SD-OCT (Spectralis HRA2, Heidelberg Engineering), SW-AF (Spectralis HRA2, Heidelberg Engineering), and wide-angle color fundus image using an Optos 200Tx unit (Optos). ffERG and EOG were conducted using the Diagnosys Espion Electrophysiology System. ffERG was performed according to ISCEV standards (34).

### Exome sequencing and analysis.

Genomic DNA was extracted from peripheral blood lymphocytes extracted from whole blood from the proband. Whole-exome sequencing was performed by Psomagen using the SureSelect Human All Exon V8 (Agilent). Sequencing reads were aligned to the hg19 reference genome using Burrows-Wheeler Alignment tool and processed with GATK according to the best practices recommendations (35). After variant calling, we narrowed our analyses to variants in genes previously associated with retinal disease (RetNet; https://sph.uth.edu/retnet/) at a MAF of equal to or less than 0.005 according to the gnomAD database (https://gnomad.broadinstitute.org/; accessed May 2022). We then performed functional annotation on the called variants with ANNOVAR (36) using pathogenicity scores from the dbnsfp 4.2a data set (37). Pathogenic effects on splicing were assessed using SpliceAI (38). Deletion-insertion-specific pathogenicity predictions were analyzed by SIFT_indels2 (39) and PROVEAN (40).

### Functional testing of BEST^Trp338_Asn339delinsCysTyr^.

Electrophysiological analyses were conducted 48–72 hours after transfection. Whole-cell patch-clamp recording was performed with an EPC10 patch-clamp amplifier (HEKA Electronics) controlled by Patchmaster (HEKA). Micropipettes were pulled and fashioned from filamented 1.5 mm thin-walled glass (WPI Instruments) and filled with internal solution containing 130 mM CsCl, 1 mM MgCl_2_, 10 mM EGTA, 2 mM MgATP (added fresh), and 10 mM HEPES (pH 7.4, adjusted by CsOH). The desired Ca^2+^ concentrations were obtained by adding CaCl^2^ (Ca/Mg/ATP/EGTA Calculator v1 was used to calculate CaCl2, https://somapp.ucdmc.ucdavis.edu/pharmacology/bers/maxchelator/CaMgATPEGTA-TS.htm). Series resistance was typically 1.5–2.5 MΩ, with no electronic series resistance compensation. The recipe of external solution was 140 mM NaCl, 5 mM KCl, 2 mM CaCl_2_, 1 mM MgCl_2_, 10 mM HEPES (pH 7.4, adjusted by NaOH), and 15 mM glucose. Solution osmolarity was 290~310 mOsm/L with glucose. Traces were acquired at a repetition interval of 4 seconds. Currents were sampled at 25 kHz and filtered at 5 or 10 kHz. I-V curves were generated from a group of step potentials (–100 to +100 mV from a holding potential of 0 mV). Experiments were conducted at room temperature (23°C ± 2°C). Whole-cell patch-clamp data were processed off-line in Patchmaster.

### Statistics.

Statistical analyses were performed using built-in functions in Origin and 2-tailed unpaired Student’s *t* test. *P* values of less than 0.05 were considered significant.

### Study approval.

All procedures reviewed were in accordance with the tenets of the Declaration of Helsinki. Studies were reviewed and approved by the Columbia University Institutional Review Board (protocol AAAB6560). Written informed consent was obtained from patients, as regulated by the Columbia University Institutional Review Board. Written informed consent was obtained for all clinical images published with this manuscript.

## Author contributions

SHT, TY, and RA designed the research studies. JW and TY conducted the experiments. MK, JW, TY, WL, and RA acquired the data. MK, JKO, JW, TY, WL, and RA analyzed the data. JZ performed genetic sequencing. PYS recruited patients. JW and TY provided reagents. MK and JKO wrote and prepared the original draft. WL, LAJ, and AHK reviewed and edited the manuscript.

## Supplementary Material

Supplemental table 2

## Figures and Tables

**Table 1:**
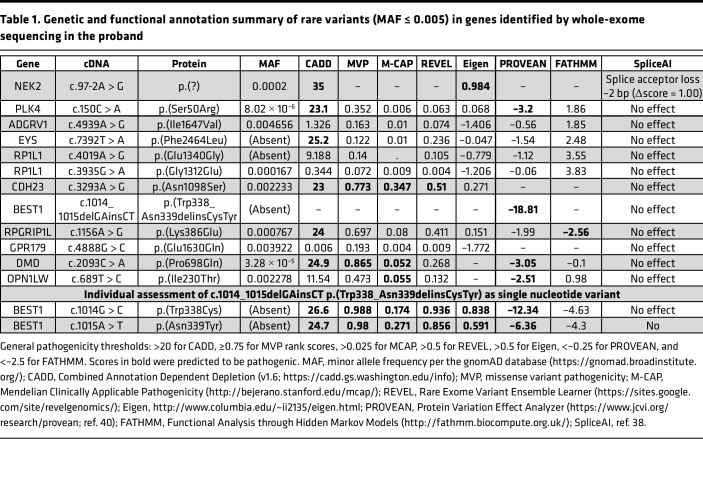
Genetic and functional annotation summary of rare variants (MAF ≤ 0.005) in genes identified by whole-exome sequencing in the proband

## Version 1 10/20/2022

In-Press Preview

## Version 2 12/08/2022

Electronic publication
