# Supplementary material for: A pathogenic in-frame deletion-insertion variant in BEST1 phenocopies Stargardt disease
Source: JCI Insight. 2022 Dec 8;7(23):e162687. doi: 10.1172/jci.insight.162687 (PMC9746905; doi:10.1172/jci.insight.162687)
Supplement: Supplemental table 2 [file jciinsight-7-162687-s246.pdf]

Supplemental Table 1. Genetic and functional annotation summary of all \variants (MAF ≤ 0.1) in the *ABCA4* gene identified by whole exome sequencing in the proband.

Gene	cDNA	Protein	MAF	CADD	MVP	M-CAP	REVEL	Eigen	PROVEAN	FATHMM	SpliceAI	SPIDEX
ABCA4	c.6816+28G>C	p.(?)	0.0730	-	-	-	-	-	-	-	no effect	1.357
ABCA4	c.6764G>T	p.(Ser2255Ile)	0.0813	11.13	-	-	0.2	-1.111	-0.99	0.348	no effect	1.71
ABCA4	c.6730-3T>C	p.(?)	0.0731	-	-	-	-	-	-	-	no effect	2.924
ABCA4	c.6729+61G>A	p.(?)	0.0120	-	-	-	-	-	-	-	no effect	-0.298
ABCA4	c.6282+7G>A	p.(?)	0.0819	-	-	-	-	-	-	-	no effect	-0.193
ABCA4	c.6249C>T	p.(Ile2083Ile)	0.0820	-	-	-	-	-	-	-	no effect	-1.057
ABCA4	c.1761-50G>A	p.(?)	0.0139	-	-	-	-	-	-	-	no effect	0.123

General pathogenicity thresholds: >20 for CADD (v1.6), ≥0.75 for MVP rank scores, >0.025 for MCAP, >0.5 for REVEL, >0.5 for Eigen, < -0.25 for PROVEAN, < -0.25 for FATHMM and >5 for SPIDEX. Abbreviations: MAF, minor allele frequency according to the gnomAD database (https://gnomad.broadinstitute.org/).
